# Development of an automated, high-throughput SARS-CoV-2 neutralization assay based on a pseudotyped virus using a vesicular stomatitis virus (VSV) vector

**DOI:** 10.1080/22221751.2023.2261566

**Published:** 2023-09-28

**Authors:** Ziteng Liang, Xi Wu, Jiajing Wu, Shuo Liu, Jincheng Tong, Tao Li, Yuanling Yu, Li Zhang, Chenyan Zhao, Qiong Lu, Haiyang Qin, Jianhui Nie, Weijin Huang, Youchun Wang

**Affiliations:** a Division of HIV/AIDS and Sex-transmitted Virus Vaccines, Institute for Biological Product Control, National Institutes for Food and Drug Control (NIFDC), WHO Collaborating Center for Standardization and Evaluation of Biologicals, NHC Key Laboratory of Research on Quality and Standardization of Biotech Products and NMPA Key Laboratory for Quality Research and Evaluation of Biological Products, Beijing, People’s Republic of China; b Chinese Academy of Medical Sciences & Peking Union Medical College, Beijing, People’s Republic of China; c Changping Laboratory, Beijing, People’s Republic of China; d Beijing Yunling Biotechnology Co., Ltd., Beijing, People’s Republic of China

**Keywords:** SARS-CoV-2, VSV, pseudovirus, neutralization assay, high-throughput

## Abstract

The global outbreak of COVID-19 has caused a severe threat to human health; therefore, simple, high-throughput neutralization assays are desirable for developing vaccines and drugs against COVID-19. In this study, a high-titre SARS-CoV-2 pseudovirus was successfully packaged by truncating the C-terminus of the SARS-CoV-2 spike protein by 21 amino acids and infecting 293 T cells that had been stably transfected with the angiotensin-converting enzyme 2 (ACE2) receptor and furin (named AF cells), to establish a simple, high-throughput, and automated 384-well plate neutralization assay. The method was optimized for cell amount, virus inoculation, incubation time, and detection time. The automated assay showed good sensitivity, accuracy, reproducibility, Z’ factor, and a good correlation with the live virus neutralization assay. The high-throughput approach would make it available for the SARS-CoV-2 neutralization test in large-scale clinical trials and seroepidemiological surveys which would aid the accelerated vaccine development and evaluation.

## Introduction

COVID-19, which is caused by SARS-CoV-2, is still a serious threat to human health, with millions continuing to be infected and thousands still dying. The neutralizing antibody against SARS-CoV-2 is an essential indicator of protective effects, whether it is from natural infection or vaccine immunity [1]. The higher the level of neutralizing antibodies, the better the protection provided. Many vaccines for SARS-COV-2 have been developed and some are in use in clinical practice [2, 3]. The levels and duration of protection of the neutralizing antibodies should be evaluated to monitor breakthrough infections and determine vaccine efficacies [4, 5]. Thus, there is a need to develop a high-throughput and simple-to-operate method to detect neutralizing antibodies in patients or vaccinated individuals.

The main approaches for SARS-CoV-2 antibody detection reported in the literature are mainly the authentic virus plaque reduction neutralization test (PRNT), pseudovirus-based neutralization assay (PBNA), and enzyme-linked immunosorbent assay (ELISA) [6]. The authentic virus plaque reduction neutralization test was developed by Henderson and Taylor in 1959 to measure the activity of neutralizing antibodies against arboviruses and it is the gold standard for the serological and immunological detection of neutralizing antibodies [7]. This method requires the use of an authentic virus, needs to be performed in a biosafety level (BSL) 3 laboratory for SARS-CoV-2, and cannot be used in high-throughput screening. Hence, PRNT is unsuitable for large-scale implementation in large-scale vaccine evaluations or seroepidemiological analyses [8, 9]. The enzyme-linked immunosorbent assay is currently considered to be a serodiagnostic method for SARS-CoV-2 infection and is mostly used to measure the level of binding antibodies in SARS-CoV-2 infections. ELISA mainly includes direct ELISA, indirect ELISA, and competitive ELISA. Competitive ELISA is usually used as a high-throughput alternative means to detect neutralizing antibodies against SARS CoV-2 [5]. However, the competitive antibodies tested in this assay are just part of the functional neutralizing antibodies, which might under-estimate the potency of the neutralization samples.

Due to their safety and easy-to-handle, pseudoviruses are becoming more widely employed in neutralizing antibody detection. Data have shown that a pseudovirus-based neutralization test correlated well with the authentic virus test method [10, 11]. The VSV pseudovirus system are divided into two categories according to the encapsulated reporters: the chemiluminescence-based and the fluorescence-based VSV system pseudoviruses. The detection steps for the chemiluminescence pseudovirus neutralization test are cumbersome, requiring the addition of exogenous substrates to generate the detectable signals, while the fluorescent pseudovirus neutralization assay could be read using the florospot reader without the addition of exogenous substrates [12]. However, high-throughput screening cannot be efficiently achieved in a manual manner. Therefore, developing a 384-well plate fluorescent pseudovirus neutralization test method using the VSV system is desirable. The fluorescent VSV system makes the fast, simple, automatic, and high-throughput neutralization test available, which would provide technical support for the quantitative determination of neutralizing antibodies in large-scale sample detections.

In this communication, we have established a 384-well plate fluorescent pseudovirus neutralization test method based on the VSV system and systemically validated this assay. The results generated in this assay are closely related to the chemiluminescence pseudovirus neutralization test, with the advantages of high-throughput screening, simple operation, and small sample volumes required for the detection of SARS-CoV-2 neutralizing antibodies.

## Materials and methods

### Cells, serum, and plasmids

293 T cells [American Type Culture Collection (ATCC), CRL-3216] were supplemented with 100 U/mL penicillin–streptomycin solutions (GIBCO), and 10% fetal bovine serum (FBS, Pansera ES, PAN-Biotech) and incubated at 37 °C and 5% CO_2_. ACE2 receptors were stably transfected into 293 T cells and screened with 15 µg/ml blasticidin to obtain 293T-ACE2 cells. The above-mentioned cells were passaged every 2–4 days. The furin receptors were stably transfected into 293T-ACE2 cells to obtain AF cells (constructed in our laboratory), using 150 µg/ml hygromycin B, 10% FBS, and 100 U/ml penicillin–streptomycin solutions. Transient transfection in 293 T cells: when 293 T cells converge to about 80% in T25 bottles, use lipo3000 transfection reagent to transfect 10 µg/T25 plasmid, place them in an incubator at 37 °C with 5% CO_2_, after 4-6 h, use DMEM with 10% FBS and 100U/mL penicillin–streptomycin to change the solution, and collect the cells 24 h later.

Positive serum samples from convalescent COVID-19 patients were collected from March to October 2020 at the Institute of Translational Medicine. 100 negative serum samples were collected from plasma donors in Shandong, Huan Jiang, Guangxi before 2020; the donors were aged 18–55 years, an average of 40 years old, with a male-to-female ratio of 1:1.3[13]. To obtain SARS-CoV and MERS-CoV positive serum samples, spike expressing plasmids derived from the two viruses were used to immunize 4 guinea pigs, respectively. Each animal was immunized with 200 ng plasmids twice at a 2-week interval. Serum samples were collected two weeks after the last jabs as the test samples for SARS-CoV and MERS-CoV, respectively.

Plasmids used in the experiment have different labels. Flag-tagged ACE2 protein (NM_021804.2) was cloned to pCMV3 with a blasticidin selection marker (E. coli, sinnobiological, China). GFP-tagged furin protein (NM_001289823.1) was cloned to pCMV3 with hygromycin B selection marker (E. coli, sinnobiological; China). GFP-tagged TMPRSS2 protein (NM_001135099.1) was cloned to pLV with a puromycin selection marker (E. coli, sinnobiological; China). GFP-tagged CTSL protein (NM_001912.5) was cloned to pLV with a puromycin selection marker (E. coli, sinnobiological; China). The empty vector pcDNA3.1 (MN996867) was from General Biology Ltd.

### Pseudovirus packaging

**Figure 1.**
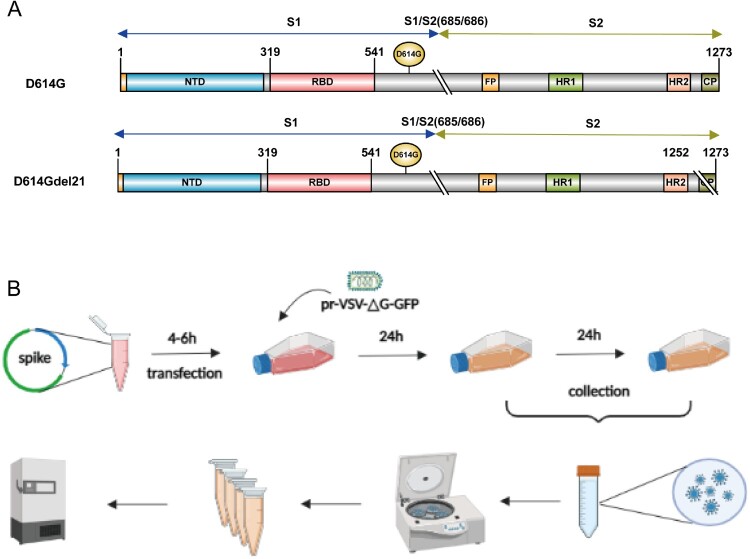
(A) Schematic diagram of D614G and D614G with a C-terminal truncation of 21 amino acids. The numbers in the figure represent the positions of amino acids; (B) SARS-CoV-2-Sdel21-GFP pseudovirus packaging flow chart. The figure is drawn using biorender. (https://app.biorender.com/).

To rescue a prVSV-ΔG-GFP pseudovirus, 293 T cells pre-plated in a six-well plate were infected with a vTF7-3 poxvirus (MOI = 5) and incubated at 37 °C and 5% CO_2_ for 1 h. GFP backbone plasmid (purchased from Kerefast) and helper plasmids (PBS-N, PBS-P, PBS-G, and PBS-L) were transfected at 5, 3, 5, 8, and 1 μg/well, using lipofectamine 3000. Then, 4–6 h after transfection, the medium was replaced with 2% DMEM medium, and the prVSV-△G-GFP pseudovirus was harvested by culturing in a 37 °C, 5% CO_2_ incubator for 24 h. The spike plasmid was obtained by cloning the codon-optimized D614G (GISAID: EPI_ISL_766872) into a pcDNA3.1 vector, named pcDNA3.1-SARS-CoV-2-D614G. Using this method, the C-terminus of the virus was truncated by from 18 to 24 amino acids to obtain a series of C terminus truncated plasmid (Figure 1A).

To package the pseudovirus, the S plasmid was first transfected in 293 T cells using lipofectamine 3000 for 4–6 h. Then, the cells were infected with the prVSV-△G-GFP pseudovirus (MOI = 0.2) for 1–2 h. The medium was changed to 2% DMEM, the supernatant containing the pseudovirus was harvested after 24 h incubation. Fresh 2% DMEM was added, and the supernatant was harvested again within 24 h and centrifuged at 4000 rpm at 4 °C for 20 min. After centrifugation, the combined supernatant of the two harvests were aliquoted and stored at –80 °C until use (Figure 1B).

### Pseudovirus titration

First, an INTEGRA VIAFL0384 instrument was used to seal the edges of a 384-well plate with sterile water (80 µl per well) to prevent the edge effect caused by the evaporation of the internal liquid. Then, a 96-well U-shaped plate was used to dilute the harvested pseudovirus in rows A–G. And the pseudovirus supernatant was added to the first column, then the pseudovirus was vertically diluted two folds. After a total of seven dilutions, an INTEGRA ASSIST PLUS pipetting workstation was used to transfer the liquid from the U-plate to a 384-well plate. The INTEGRA ASSIST PLUS instrument was used to automatically add 2 × 10^3^ cells/well of AF cells (20 µl per well), and incubated at 37 °C and 5% CO2 for 24 h. Then the fluorescent spot counter (Cytation 5, Biotek) was uesd to count the number of GFP-positive cells to calculate the SARS-CoV-2-S-GFP pseudovirus titres (focus-forming units per ml (FFU/ml)) [14].

### Pseudovirus neutralization

In a 96-well U-shaped plate, the samples to be tested in wells A2–G11 were diluted, 10 samples were added to each plate, 15-fold dilution was performed in the first row, and the INTEGRA ASSIST PLUS pipetting workstation was used for 3-fold gradient dilution, a total of seven samples. After the dilution, the INTEGRA ASSIST PLUS pipetting workstation was used to transfer the samples to be tested in the U-shaped plate to a 384-well plate, and two duplicate wells were set up, each 10 µl. The pseudovirus was diluted with DMEM complete medium to approximately 400 GFP-positive particles per well, and 10ul of pseudovirus was added to wells B3-O23. While the plates were incubated for 1 h at 37 °C and 5% CO_2_, the cells were diluted to 1 × 10^5^ cells/ml suspension, and were added 20 µl to each well (2 × 10^3^ cells/well). Then, the 384-well plate was placed in a 37 °C, 5% CO_2_ incubator for 24 h. After incubation, the number of GFP-positive cells were counted by an immunospot reader (Biotek) to calculate the 50% of neutralizing titre (NT_50_) using the Reed-Muench method.

### Authentic virus neutralization assay

Authentic virus neutralization was performed as described below. 50 ul of serially diluted monoclonal antibodies were added to 96-well plates. Then, 50 ul of SARS-CoV-2 authentic viruses were added with a concentration of 2000 CCID50/ml. After 1-hour incubation at 37 °C and 5% CO_2_, 100 µl Vero cells with a concentration of 2 × 10^5^ cells/ml were added to the plates. After 5 d of incubation at 37 °C with 5% CO_2_, the cytopathic effect (CPE) of each well was observed under microscopes by three different individuals. Next, the EC_50_ was calculated using Reed-Muench [8]. This assay was performed in a biosafety level (BSL) 3 laboratory.

### Animal immunization

Female Guinea pigs (weights 200–220 g) were used as the experimental animals and were divided into six groups with nine animals in each group. Guinea pigs were immunized with D614G, Alpha, Beta, Gamma, Delta, and Omicron proteins. Spike protein (100 µg) was mixed with Al adjuvant and the guinea pigs were immunized three times at 14-day intervals. Serum was collected 14 days after the third immunization for subsequent experiments. Female guinea pigs (weight 200–220 g) without immunization were used as the negative test animals and 100 negative animal serum samples were obtained.

### Statistical analysis

The paired comparisons were conducted using student’s t-test. A paired x2 test (McNemar’s x2test) and kappa values were used to assess the difference in qualitative results obtained from 384-well plate SARS-CoV-2-Sdel21-GFP pseudovirus neutralization assay and 96-well SARS-CoV-2-Sdel21-Fluc pseudovirus neutralization assay. Pearson’s correlation coefficient was employed to analyze the strength of the linearity. To compare the quantitative results obtained for the positive samples detected by both assays, a fitted regression model was compared by testing the two-tailed hypothesis of slope 1 and intercept 0. Bland–Altman method, i.e. a scatter plot of the differences between the paired measurements against the mean values of the samples, was used to assess the magnitude of disagreement between the two assays [15, 16]. Data were analyzed with GraphPad Prism 8.0 software (GraphPad, San Diego, CA). The results are presented as the means § standard deviations (SD). Significance thresholds: * *p* < 0.05, ** *p* < 0.01, *** *p* < 0.005, and **** *p* < 0.001. The formula for calculating the Z-value is [17].

## Results

### Optimization of the conditions to achieve high-titre pseudoviruses

#### Spike plasmid of SARS-CoV-2

**Figure 2.**
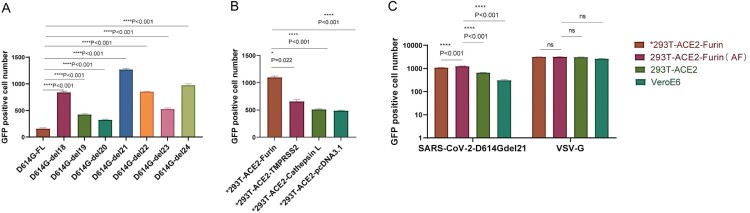
Optimization of the S protein plasmids and selection of sensitive cells. (A) The investigation of the effects of truncation of amino acids from 18 to 24 at the C terminus of the spike plasmid of D614G on the pseudovirus titres; (B) Transfection of furin, TMPRSS2, cathepsin L and pcDNA3.1 using transient transfection of ACE2 in 293 T cells. The above plasmids were transiently transfected for a single time according to 10 µg of plasmid in each T25 bottle (if two plasmids were transfected at the same time, the total plasmid amount was 10 µg). Cells were harvested 24 h later for a cell sensitivity test. The number of GFP-positive cells after virus infection in different cells was counted with the Biotek instrument; (C) Comparison of the infection efficiency of SARS-CoV-2-D614Gdel21-GFP in 293 T cells that were transiently transfected with ACE2 and Furin (*293T-ACE2-Furin), 293 T cells which were stably transfected ACE2 and Furin (293T-ACE2-Furin), named AF, 293T-ACE2, and VeroE6 cells. All data are obtained through three independent repeated experiments. The bar value represents the mean with SD. Cells with * are represented by transiently transfected cells.

It has been reported that the SARS-CoV-S protein variant with a cytoplasmic tail truncation of 18 [18], 19 [19], or 24 [20] amino acids could enhance the packaging efficiency and yield relatively high-titre pseudoviruses. To distinguish the highest-efficiency modification, we truncated the C-terminus of the SARS-CoV-2 D614G spike protein from 18 to 24 amino acids and then packaged pseudoviruses, respectively (Figure 2A). The titration results showed that the pseudovirus titres were increased to varying degrees compared to the full-length spike protein, and the truncation of 21 amino acids showed the most significant effect. The pseudovirus titre with D614Gdel21 (named SARS-CoV-2-Sdell21-GFP) was approximately 8 times higher than the untruncated D614G.

#### Sensitive cells

Besides the angiotensin-converting enzyme 2 (ACE2) receptor, a series of proteinases have been reported to play different roles in the infection of SARS-CoV-2. To identify the sensitive cells to the SARS-CoV-2 infection, angiotensin-converting enzyme 2 (ACE2) (NM_021804.2), furin (NM_001289823.1), type II transmembrane serine protease (TMPRSS2) (NM_001135099.1), cathepsin L (NM_001912.5), and empty vector pcDNA3.1 (MN996867) were studied for their assistance in virus infections. The cells were co-transfected with the ACE2 receptor and furin, type II transmembrane serine protease (TMPRSS2), or cathepsin L. The transfection efficiencies were tested using flow cytometry. We found that the proportion of co-transfection of two plasmids was approximately 30% (Supplementary Figure S1). The titration results of SARS-CoV-2-D614Gdel21-GFP fluorescent pseudovirus in the above cells showed that the number of positive cells transiently transfected with both the ACE2 and furin was the highest (Figure 2B). Compared to the transiently transfected 293 T cells with ACE2 and furin, 293 T cells with ACE2, and VeroE6 cells, the AF cells showed the highest fluorescence signals (Figure 2C). Therefore, we isolated the stably transfected cells with both the ACE2 and furin as the target cells for the following study, which we named AF cells.

### Optimization of the key parameters for the neutralization assay

#### Cell addition

**Figure 3.**
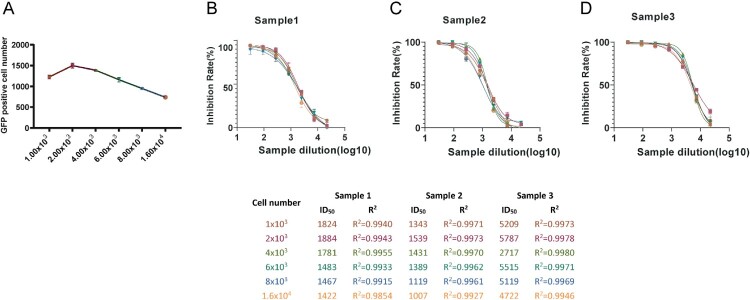
Optimization of cell number. (A) Optimization of the number of cells added in the pseudovirus titration assay; (B-D) To optimize the seeding cell number, 1 × 10^3^–1.6 × 10^4^ cells/well were added and the cells were infected with an inoculant dose of 0.2 MOI, and the NT_50_ values of Sample 1, Sample2 and Sample 3 were calculated with non-linear regression, i.e. log(inhibitor) vs. response (four parameters). All data are obtained through three independent repeated experiments.

To optimize the number of cells in the SARS-CoV-2-D614Gdel21-GFP titration and neutralization test, a triad of representative positive samples, namely monoclonal antibody AM180 (Sample 1); convalescent COVID-19 patient serum (Sample 2); and guinea pig D614G spike protein of SARS-CoV-2 immunized three times serum (Sample 3) were employed. To determine the optimal cell seeding amount, titration and neutralization experiments were conducted by adding 1.00 × 10^3^–1.6 × 10^4^ cells/well. The titration outcomes revealed that the highest number of GFP-positive cells were observed at 2.00 × 10^3^ cells/well (Figure 3A). A four-parameter curve was fitted to determine the inhibition rate at various dilutions (log_10_), yielding R^2^ values > 0.9, indicating a good curve of fit (Figure 3B-D). The highest 50% neutralization titres (NT_50_) values were obtained when 2.00 × 10^3^ cells/well were added. Based on these findings, 2.00 × 10^3^ cells/well was deemed the optimal cell seeding amount.

#### Virus inoculation

**Figure 4.**
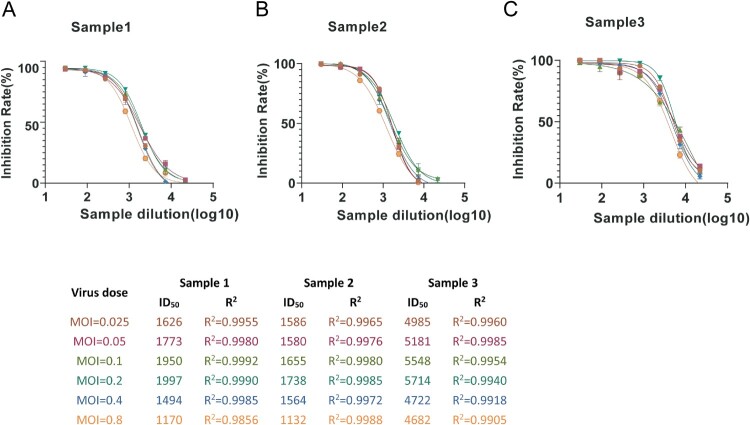
Optimization of pseudovirus inoculated dose. (A-C) The optimal inoculant dose of the pseudovirus was investigated using doses from 0.025–0.8 MOI. The amount of cell addition were 2.00 × 10^3^ cells/well, and the NT_50_ values were calculated with non-linear regression, i.e. log(inhibitor) vs. response (four parameters). All data are obtained through three independent repeated experiments.

To determine the optimal amount of virus in this assay, we set the MOI of the virus to be in the range of 0.025–0.8, calculated the results for the samples at different dilutions (log_10_), and plotted the four-parameter curve of the inhibition rate (Figure 4A-C). The R^2^ values were all higher than 0.99, indicating that the curve fitted well. When the virus MOI = 0.2, the sample NT_50_ values tended to be the highest and most stable. When the virus inoculum was higher or lower than the MOI = 0.2, the NT_50_ values showed a downward trend. Therefore, the optimal amount of added virus was determined to be MOI = 0.2, and the number of GFP-positive cells was approximately 400 in the virus control well of the 384-well plate.

#### Pre-incubation time

**Figure 5.**
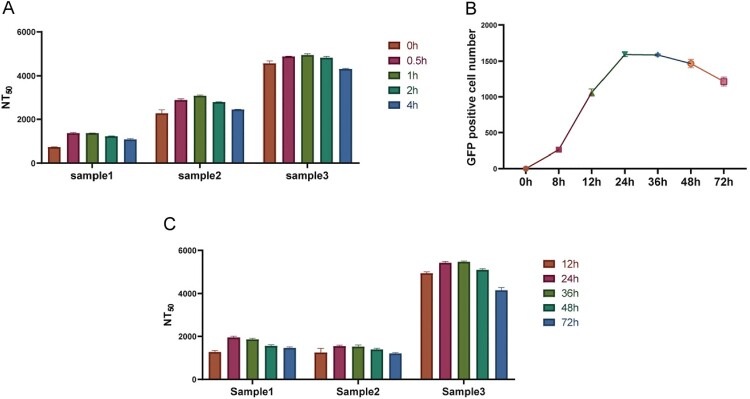
Optimization of pre-incubation and incubation time for pseudovirus neutralization assay. (A) The effect of different pre-incubation times on the NT50 values in the SARS-CoV-2-D614Gdel21-GFP fluorescent pseudovirus neutralization assay; (B) The number of GFP-positive cells detected at 0, 8, 12, 24, 36, 48, and 72 h were compared in the pseudovirus titration test; (C) Comparison of the NT50 values of Sample 1, Sample 2, and Sample 3 at the end of different incubation times. All data are obtained through three independent repeated experiments. The bar value represents the mean with SD.

For the virus neutralization assay, the virus and serum samples are usually pre-incubated to ensure the antibodies completely binding to the viruses before being added to the target cells [9, 21]. To determine the optimal pre-incubation time for the samples with the pseudovirus in the neutralization test, the pre-incubation time was set as 0, 0.5, 1, 2, and 4 h to investigate the effects of the different pre-incubation times on the results of the pseudovirus-based neutralization assay (Figure 5A). The results showed that when the pre-incubation time was 0.5–2 h, there was no significant difference in the detected NT_50_ values. When the pre-incubation time was 2 h or longer, the NT_50_ values would decrease dramatically. Therefore, we determined that the optimal pre-incubation time was 1 h, which could be altered in the range of 0.5–2 h.

#### Incubation time

To determine the optimal detection time, we detected the number of GFP-positive cells after 0, 8, 12, 24, 36, 48, and 72 h of incubation for the fluorescent pseudovirus SARS-CoV-2-Sdel21-GFP with the cells at 37 °C in the titration test (Figure 5B). The NT_50_ values of three representative samples at different times in the neutralization assay were determined (Figure 5C). The results showed that the number of GFP-positive cells increased rapidly at 8–24 h after infection and remained stable between 24–36 h. The neutralization test results at different times showed that the sample NT_50_ value at 24–36 h was relatively stable. Because pseudoviruses cannot produce progeny viruses, the number of cells infected will be a good indicator of infection efficiency. Therefore, by counting the number of GFP-positive cells at 24 h, the infection and neutralization efficiency of the pseudovirus can be quantified.

#### Establishment of an automatic pseudovirus neutralization test based on the VSV system in a 384-well plate pattern

**Figure 6.**
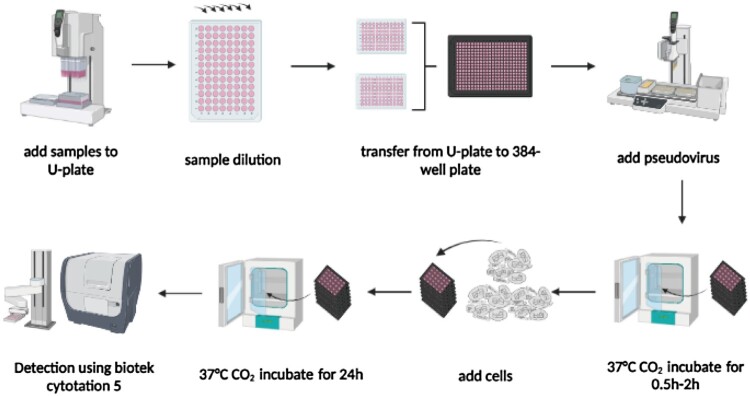
Establishment of the automatic fluorescent pseudovirus neutralization test based on the VSV system in a 384-well plate. This figure is drawn using biorender.

The dilution and addition of the samples, cells, and pseudoviruses, and the detection of GFP fluorescent spots were integrated into an automatic process in this neutralization assay (Figure 6). Briefly, the samples to be tested were added and diluted in the 96-well U-shaped dilution plate using the INTEGRA automatic sample processing asset. Then, the diluted samples of the two 96-well U-plates were transferred to a 384-well plate using an INTEGRA ASSIST PLUS pipetting station. Next, the fluorescent pseudovirus was diluted to yield approximately 400 GFP-positive spots per well, and automatically added into the 384-well plate with 10 µl of the pseudovirus in each well. The 384-well plate was then incubated at 37 °C with 5% CO_2_ for 0.5–2 h. The AF cells were prepared and adjusted to 1.00 × 10^5^ cells/ml, and automatically added to each well with a volume of 20 µl, followed by culturing in a 5% CO_2_ incubator at 37 °C for 24–36 h. After incubation, the 384-well plates were transferred to a florospot reader (Biotek Cytation 5) equipped with a robotic arm to automatically count the number of GFP fluorescent spots. Finally, the data were directly imported into a table with an algorithm, and the NT_50_ values were calculated using the Reed-Muench method to indicate the levels of neutralizing antibodies in the samples.

### Method validation

**Figure 7.**
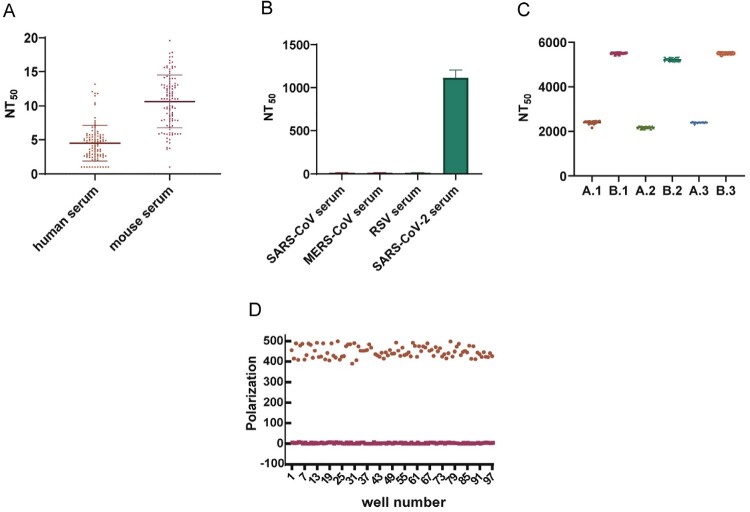
Methodological validation of the fluorescent pseudovirus 384-well plate neutralization assay. (A) Sensitivity of the pseudovirus assay; (B) Specificity of the pseudovirus assay; (C) Reproducibility of the pseudovirus assay; (D) The number of GFP-positive cells was obtained by analyzing 100 positive wells and 100 negative wells using the 384-well plate fluorescent pseudovirus neutralization assay. All data are obtained through three independent repeated experiments. The bar value represents the mean with SD.

To determine the cutoff value of this neutralization assay, 100 human negative sera and 100 guinea pig negative sera samples were initially diluted 2-fold, followed by 2-fold serial dilutions to calculate the NT_50_ (Figure 7A). The confidence interval (mean ± SD) of the human negative serum samples was 4.49 ± 2.62 and the confidence interval (mean ± SD) of the guinea pig negative serum samples was 10.62 ± 3.87; the detection limit = mean + 1.96 SD, so the limits of quantification for human serum and guinea pig serum were 9.61 and 18.21, respectively. The cutoff values for the final human and mouse serum samples were set at 10 and 20, respectively. Based on the set cutoff value, the specificity of human negative sera and guinea pig negative sera were 97.0% and 100.0%, respectively. Then, we used the SARS-CoV-2-D614Gdel21-GFP fluorescent pseudovirus neutralization assay to test four severe acute respiratory syndrome coronavirus (SARS-CoV) antibody-positive sera, four middle east respiratory syndrome coronavirus (MERS-CoV) antibody positive sera, four respiratory syncytial virus (RSV) antibody positive sera, and four SARS-CoV-2-antibody positive sera (Figure 7B). While the SARS-CoV, MERS-CoV, and RSV antibody-positive sera could not neutralize SARS-CoV-2-D614Gdel21-GFP fluorescent pseudovirus, only the SARS-CoV-2 antibody-positive sera showed neutralization against the SARS-CoV-2-D614Gdel21-GFP fluorescent pseudovirus, suggesting the reasonable specificity for this approach.

To determine the sensitivity of this method, we simultaneously used 384-well plate SARS-CoV-2-Sdel21-GFP pseudovirus neutralization assay with the existing 96-well SARS-CoV-2-Sdel21-Fluc pseudovirus neutralization test to test the first-generation international standard (NIBSC code: 20/136, 1000 IU/ml). After calibration with the international standard, the cutoff values for human and mouse sera were 5.18 and 10.36 IU, respectively.

To determine the accuracy of this method, we conducted spike-and-recovery experiments using a negative human serum sample (NT50 < 20). We added monoclonal antibody AM180 at concentrations of 1:1, 1:5, and 1:25, respectively, and measured the NT50 values of the negative guinea pig serum and the spiked sample. The recovery rates for the three addition were 105.3.%, 100.4%, and 99.8%, respectively, indicating a good accuracy.

To investigate the reproducibility of this assay, two serum samples from guinea pigs were employed, which were obtained from guinea pigs immunized with three doses of the spike protein. The tests were repeated three times at different times, and 20 replicates of the samples were tested in each experiment (Figure 7C). The intra-assay coefficient of variation (CV) values were between 0.4% and 3.0%, and the inter-assay variability CV values were between 1.7% and 6.2%, indicating good reproducibility for this assay.

The Z ‘factor is widely used in the HTS test to represent the quality of the measured samples [17, 22]. To determine whether this assay is suitable for high-throughput detection, we calculated the Z ‘factor for this assay. The average polarization values were approximately 448.5 ± 33.8 and 2.7 ± 0.8 in negative controls and positive controls, respectively (Figure 7D). The Z’ factor for this assay was 0.77, which indicates that it could be used as a robust high throughput neutralization assay.

**Figure 8.**
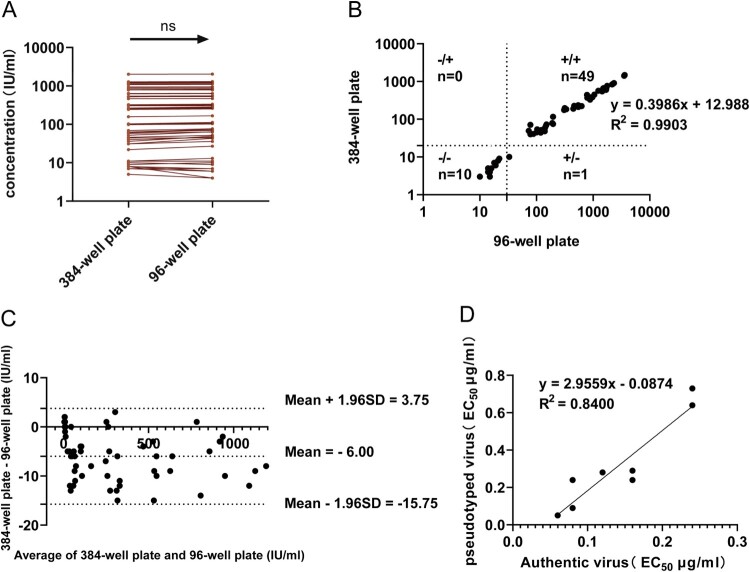
Comparison of the 384-well plate GFP pseudovirus assay, 96-well plate Fluc and live virus assay. (A) NT_50_ values were obtained by analyzing 60 guinea pig serum samples using the two neutralization test methods (384-well plate fluorescent pseudovirus neutralization assay and 96-well plate Fluc pseudovirus neutralization assay) after calibration with international standards for consistency comparison; (B) Correlation between the 384-well plate and the 96-well plate neutralization assay for comparison of all the 60 samples. The qualitative comparison of the results from the two assays showed concordance of 49 positive and 10 negative samples (98.0%, Kappa = 0.942). The fitted regression line is presented by the equation y = 0.3986x + 12.988, R^2^ = 0.9903; (C) Agreement between the 384-well plate and the 96-well plate neutralization assay for 50 positive samples and 10 negative samples analyzed by Bland-Altman plot; (D) Correlation analysis of EC_50_ values between the 384-well plate pseudovirus neutralization assays and authentic neutralization assay. The fitted regression line is presented by the equation y = 2.9559x - 0.0874, R^2^ = 0.84.

When a first-generation international standard (NIBSC code: 20/136, 1000 IU/ml) was employed as the calibration sample, fifty guinea pig positive sera and ten guinea pig negative sera were tested both in the 384-well plate GFP pseudovirus neutralization assay and the 96-well plate Fluc pseudovirus neutralization test. No significant differences were found in the two assays when tested with the same samples (*P* > 0.05) (Figure 8A). To further determine the correlation between 384-well plate SARS-CoV-2-Sdel21-GFP pseudovirus neutralization assay and 96-well SARS-CoV-2-Sdel21-Fluc pseudovirus neutralization assay in qualitative analyses of SARS-CoV-2-specific sera samples. Among the 60 samples, 49 (98.0%) were identified as positive by 96-well assay and 49 (98.0%) by 384-well assay, respectively. 10 (100%) were identified as negative by 96-well assay and 10 (100.0%) by 384-well assay, respectively. (Figure 8B). The qualitative comparison of the results from the two assays showed concordance of 49 positive and 10 negative samples (98.0%, Kappa = 0.942). Further analysis revealed that the neutralizing antibody values of the sample were determined as 29 by 96-well assay, and 8 by 384-well assay; the values were close to the cutoff value, indicating discordance was mainly associated with low titres of the serum samples. The correlation between the two assays was also determined in quantitative analyses. The linear equation was obtained as y = 0.3986x + 12.988, R^2^ = 0.9903 (X-axis is the NT_50_ value of the chemiluminescence system, Y-axis is the NT_50_ value of the fluorescence system), indicating that the fluorescent 384-well plate SARS-CoV-2-S-GFP pseudovirus neutralization assay established in this study had a good correlation with previously reported SARS-CoV-2-S-Fluc pseudovirus neutralization assay [9, 10]. Then, to confirm the correlation between the two assays, we employed the Bland–Altman model [15], the differences of NT_50_ values after correction by the international standard between each sample (the authentic virus neutralization assay-the pseudotyped virus neutralization assay) were plotted against the mean values obtained by the two assays (Figure 8C). The mean of NT_50_ was −6.00, with the SD of NT_50_ being 4.97. Based on the Bland–Altman plot, the limits of agreement were −15.75 and 3.75 (mean ± 1.96 SD), revealing that the differences for all 60 samples determined by both assays fall within the limits of agreement.

Nine monoclonal antibody samples were tested with the 384-well plate fluorescent pseudovirus neutralization assay and authentic neutralization assay. The EC_50_ values of monoclonal antibodies measured by the two methods showed a strong correlation with a correlation efficiency R^2^ of 0.84 (Figure 8D).

## Discussion

The neutralizing antibodies against SARS-CoV-2 are important indicators for evaluating the immune efficacy of COVID-19 vaccines. The accuracy of neutralizing antibody testing results is of great significance for the development and application of vaccines, as well as for understanding the epidemiological statuses of the convalescent individuals [23]. Currently, a variety of approaches have been employed in measuring neutralizing antibody levels against SARS-CoV-2 including authentic neutralization assay, pseudovirus neutralization assay, ELISA, etc. Although the authentic neutralization assay is considered the gold standard, it requires to be conducted under the biosafety level 3 conditions in a low throughput manner. It is urgently needed to develop accurate, easy-to-perform, and high throughput measures to iterate the neutralizing antibodies derived from the vaccinated or the infected individuals. Lots of alternative neutralizing antibody detection assays have been developed to address this issue, such as the competitive ELISA, and chemiluminescence assays [24]. However, the binding assays could not accurately indicate the functional antibodies in the samples. The development and application of the pseudovirus partially provided the resolution, which acted as a surrogate for the authentic viruses. The main shortcomings of the pseudovirus neutralization assay are the tedious operation and low-throughput manner. In this communication, we established a high-throughput and automated pseudovirus-based neutralization assay to address this issue.

Truncated modification of the C terminus of the spike protein yielded high-titre pseudoviruses to achieve a higher signal-to-noise ratio. The Spike protein of SARS-CoV-2 has a single ectodomain of 1182 aa and a transmembrane region followed by a cytoplasmic domain of 28 aa [25, 26]. It was reported that a pseudotyped VSV and a retrovirus bearing a SARS-CoV-S protein variant with a truncation in the cytoplasmic tail had much higher infection efficiency than that with full-length S protein [27]. Through comprehensive comparison, we identified the 21 amino acids deletion at the C-terminus showed the highest efficiency in mediating the SARS-CoV-2 pseudovirus infection. Another advantage of this assay was to identify an engineered cell line that over-expressed the hACE2 and Furin to assist the pseudovirus infection after systematic evaluation of the effects of different proteinases.

Compared to the 96-well plate method, the 384-well plate assay just requires one-eleventh of the sample volumes and could test 20 samples simultaneously in one plate over the four samples in a 96-well plate pattern. When considering the efficiency and cost, the 96-well plate assay can detect up to 600 samples per day, about $3.40 per sample. The 384-well plate assay can detect 5000 samples per day, about $0.48 per sample, with a 7-fold increase in efficiency and a 7-fold. decrease in cost. The cost reduction is mainly due to the direct data readout of 384-well plate GFP pseudovirus neutralization assay using florospot reader without the addition of the chemiluminescence substrate. By employing the fluorescent pseudovirus, the 384-well plates assay could be equipped with an automatic reader without adding the substrates for the chemiluminescence reporter pseudoviruses, which were integrated with the robotic liquid processing instrument to realize the automation of the neutralizing antibody detection.

During the establishment of the neutralization assay, a series of parameters including cell number, virus dose, pre-incubation time, and incubation time were optimized to achieve a robust assay. By using the automatic instruments, the high-thoughput assay showed excellent reproducibility. The Z-score analysis indicate it could be employed as a high-throughput assay. The results generated in the new method showed good correlation with the previously reported pseudovirus assay and the gold-standard live virus assay, especially when the international standards [28, 29] were introduced into the calculation of the results. It is highly recommended to use international standards, secondary standards or working references in the neutralization assay to make the results from different laboratories comparable.

In short, based on the VSV system, we have established a 384-well plate fluorescent pseudovirus neutralization assay for SARS-CoV-2 neutralizing antibody detection. This method has the advantages of high throughput, easy operation, small sample volume consumption and automation. The method would further accelerate the development and evaluation of SARS-CoV-2 vaccine and therapeutics when the emerging variants continuously challenge the current vaccines.

## Supplementary Material

Supplemental Material
