# Supplementary material for: Development of an automated, high-throughput SARS-CoV-2 neutralization assay based on a pseudotyped virus using a vesicular stomatitis virus (VSV) vector
Source: Emerg Microbes Infect. 2023 Sep 28;12(2):e2261566. doi: 10.1080/22221751.2023.2261566 (PMC10540657; doi:10.1080/22221751.2023.2261566)
Supplement: Supplemental Material [file TEMI_A_2261566_SM2145.doc]

Supplementary Materials


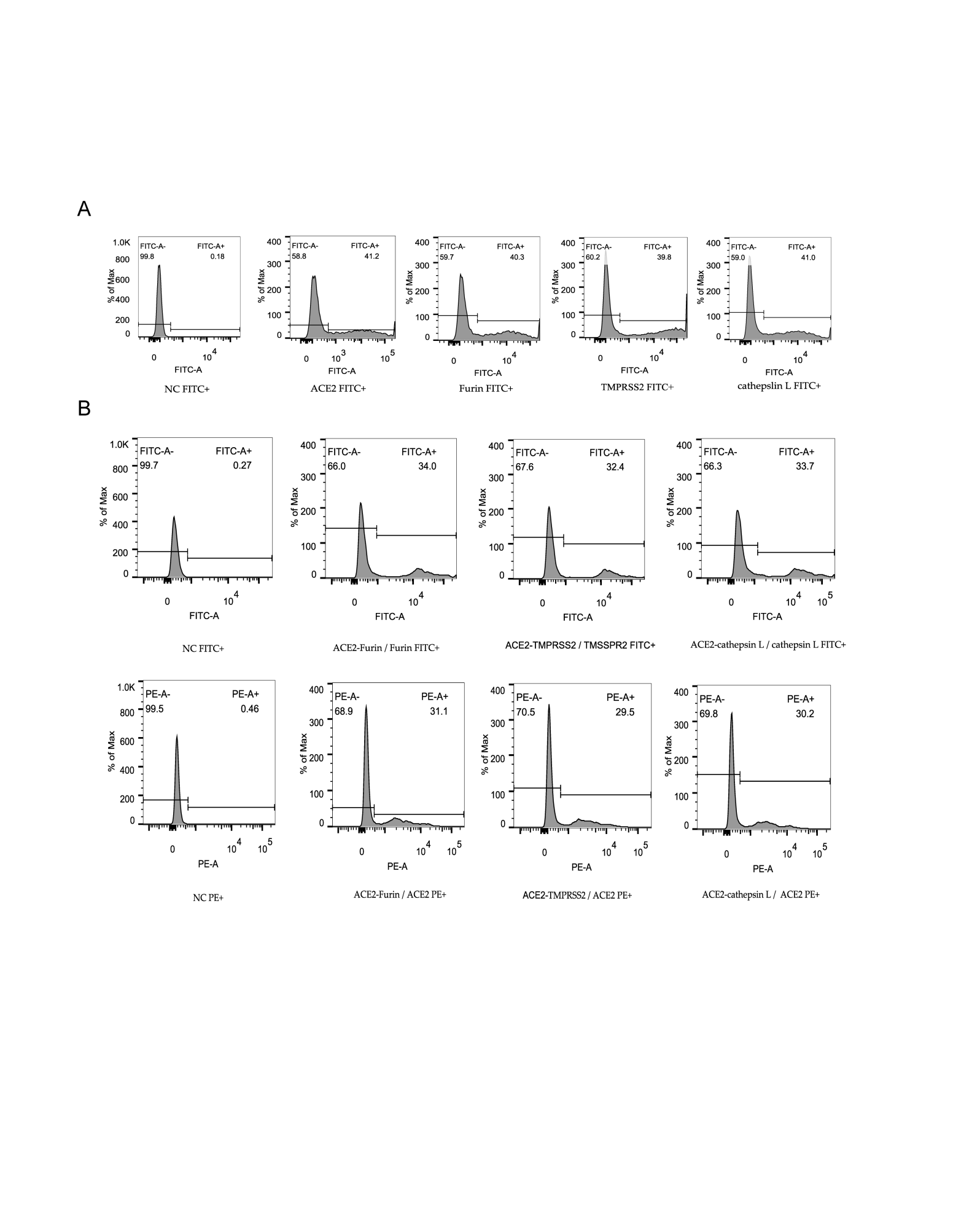


Figure S1. Cell expression of flag-tagged ACE2 and other GFP-tagged proteases is assessed by flow cytometry. (A) Surface expression levels of ACE2, furin, TMPRSS2, and cathepsin L. ACE2 were dyed with FITC to label the Flag tag. Expression of furin, TMPRSS2, and cathepsin L were assessed by the fused GFP tag using the FITC channel. (B) Surface expression levels of ACE2-Furin, ACE2-TMPRSS2, and ACE2-cathepsin L. ACE2 were dyed with PE to label the Flag tag, while the furin, TMPRSS2 and cathepsin L with fused GFP were assessed using the FITC channel. FITC-A+/PE-A+ values in the upright corner represent the percentage of cells expressing ACE2 or proteases. The average of three independent replicates is presented in the figures.

Table S2. The list of primers used for cloning

| Primers | Sequences |
| --- | --- |
| ACE2-F | ATGTCAAGCTCTTCCTGGCTCCTTCTCAG |
| ACE2-R | TAAAAGGAGGTCTGAACATCATCAGTGT |
| TMPRSS2-F | ATGCCCCCTGCCCCGCCCGGAGGTGAAA |
| TMPRSS2-R | TTAGCCGTCTGCCCTCATTTGTCGATAAA |
| Furin-F | ATGGAGCTGAGGCCCTGGTTGCTATGGGT |
| Furin-R | TCAGAGGGCGCTCTGGTCTTTGATAAAGG |
| CTSL-F | ATGAATCCTACACTCATCCTTGCTGCCTTT |
| CTSL-R | CACACAGTGGGGTAGCTGGCTGCTGAGG |
| D614G-F | ATGTTCGTGTTCCTGGTGCTGCTGCCTCTG |
| D614G-R | TTGAAAGGGTGTTGTTCGTGTGGGTCCTAA |
